# COL11A1-Driven Epithelial–Mesenchymal Transition and Stemness of Pancreatic Cancer Cells Induce Cell Migration and Invasion by Modulating the AKT/GSK-3β/Snail Pathway

**DOI:** 10.3390/biom12030391

**Published:** 2022-03-02

**Authors:** Hui Wang, Huichao Zhou, Hong Ni, Xiaohong Shen

**Affiliations:** 1 Drug Synthesis Laboratory, Tianjin Institute of Medical & Pharmaceutical Sciences, Tianjin 300020, China; 1120180544@mail.nankai.edu.cn; 2 School of Medicine, Nankai University, Tianjin 300071, China; 2120201262@mail.nankai.edu.cn (H.Z.); hongni@nankai.edu.cn (H.N.)

**Keywords:** COL11A1, pancreatic cancer, EMT and cell stemness, migration and invasion, AKT/GSK-3β/Snail signaling

## Abstract

Background: Collagen type XI α1 (COL11A1) is associated with tumorigenesis and development in many human malignancies. Previous reports indicate that COL11A1 may be a significant diagnostic marker for pancreatic ductal adenocarcinoma (PDAC); however, its biological role in PDAC progression remains unclear. In this study, we investigated the influence of COL11A1 on the invasion and migration abilities of pancreatic cancer cells and explored its potential molecular mechanisms. Methods: Cell migration and invasion were assessed using Transwell assays in pancreatic cancer cells transfected with siCOL11A1 and pCNV3-COL11A1 plasmids. The protein and mRNA expression levels of N-cadherin, E-cadherin, Vimentin, cluster of differentiation (CD)-24, CD44, serine–threonine kinase (AKT), glycogen synthase kinase (GSK)-3β, phospho (p)-AKT^Ser473^, p-GSK-3β^Ser9^, and Snail were analyzed using Western blotting and real-time polymerase chain reaction (PCR). The effect of COL11A1 on cell stemness was tested using flow cytometry and clone formation assays. Results: These results demonstrated that COL11A1 significantly promoted the invasion and migration abilities of PDAC cells. Furthermore, COL11A1 facilitated the occurrence of epithelial–mesenchymal transition (EMT) and cell stemness by upregulating the expression levels of p-AKT^Ser473^, p-GSK-3β^Ser9^, and Snail. Conclusions: This study suggests that the activation of the AKT/GSK-3β/Snail signaling pathway induced by COL11A1 plays a major role in the progression of PDAC. Therefore, COL11A1 could serve as a potential target for PDAC treatment.

## 1. Introduction

Pancreatic ductal adenocarcinoma (PDAC) is one of the most lethal solid tumors due to its propensity for early metastasis and local invasion [1,2]. Most patients with PDAC present with metastasis at the time of diagnosis and systemic chemotherapy is the main treatment, but the survival outcome is often unsatisfactory [3]. Thus, it is imperative to gain a deeper understanding of the molecular mechanisms underlying PDAC carcinogenesis and progression to identify novel diagnostic and therapeutic targets for this disease.

A histological hallmark characteristic of PDAC is the abundant extracellular matrix that constitutes its tumor microenvironment (TME). The TME is composed of a complex network of molecules with distinct biochemical properties that regulate tumor progression and metastasis. Among these components, collagen serves as a module of diverse signaling and is involved in the regulation of the physiological state in tumor cells [4].Recent evidence suggests that collagen type XI alpha 1 (COL11A1) is highly expressed in the invasive edge of pancreatic cancer tissues and is a novel biomarker associated with poor survival and chemoresistance in PDAC [5,6]. Moreover, COL11A1 is closely involved in the migration and invasion of lung and gastric cancers [7,8]. However, the molecular mechanisms of COL11A1 associated with PDAC migration and invasion remain elusive.

Epithelial–mesenchymal transition (EMT) is considered to be a vital process for inducing cancer cell metastasis [9]. During EMT, the molecular repertoire of cells, particularly E-cadherin (E-cad), vimentin (VIM), and N-cadherin (N-cad), undergoes dramatic changes. EMT can be initiated by various intrinsic and extrinsic signals, including the transforming growth factor β (TGFβ) [10], hepatocyte growth factor (HGF) [11], Snail [12], collagen I [13], and hyaluronan [14]. Moreover, studies have reported that EMT enhances the expression of stem cell markers and induces cancer cells to acquire epithelial stem cell properties, which contributes to tumorigenesis [15].

Accumulated evidence has indicated that COL11A1 modulates serine–threonine kinase (AKT) signaling pathways in various cellular events [16,17], including cell proliferation, apoptosis, metastasis, and drug resistance [16,18,19]. AKT is closely related to EMT [20,21] and is involved in many pathophysiological processes, such as angiogenesis, invasion, and metastasis [22]. Glycogen synthase kinase (GSK)-3β is the classical downstream signaling pathway of AKT and often participates in the initiation of EMT [23]. According to reports, the combination of GSK-3β inhibitors with chemotherapy is strategically poised to be a promising approach to overcome the emergence of early drug resistance or to overcome chemoresistance in advanced and metastatic pancreatic tumors [24]. The stability of Snail can be modulated by the AKT/GSK-3β signaling pathway [25,26], and activation of this pathway may further drive EMT in hepatoma [27] and ovarian carcinoma cells [28]. Furthermore, the relationship between Snail, EMT, and the cell stemness program in cancer has been investigated in several studies [29,30,31]. However, it remains unclear whether the AKT/GSK-3β/Snail signaling pathway is a key molecular cascade in the regulation of EMT and cell stemness induced by COL11A1 in PDAC.

In the present study, we elucidated the function of COL11A1 in promoting EMT and cell stemness via the AKT/GSK-3β/Snail signaling pathway, which facilitates the invasion and migration of pancreatic cancer cells. We focused on EMT and cell stemness programs to better understand the intricate molecular mechanisms by which COL11A1 regulates cancer development. This study provides valuable information that may aid in developing personalized therapeutic strategies for patients with pancreatic cancer.

## 2. Materials and Methods

### 2.1. Cell Culture, Transfection, and Treatment

PANC-1,BxPC-3, and Capan-2 cells were purchased from the American Type Culture Collection (ATCC). Capan-2 and BxPC-3 cells were cultured in the Roswell Park Memorial Institute (RPMI)-1640 medium, and PANC-1 cells were incubated in Dulbecco’s modified Eagle medium (DMEM) containing 10% fetal bovine serum (FBS; BI, Kibbutz Beit-Haemek, Israel) and 1% penicillin–streptomycin. All the cells were cultured in an incubator with 5% carbon dioxide (CO_2_) at 37 °C.

Small interfering RNAs (siRNAs) against COL11A1, GSK-3β, and Snail were purchased from Gene Pharma (Suzhou, China). The pCMV3-COL11A1 plasmid was purchased from Sino Biological (Cat. HG18256-UT; Beijing, China). The pCMV3-COL11A1 plasmid and siRNAs were transfected into cells using Lipofectamine 2000 (Invitrogen, Carlsbad, CA, USA). LY294002, an inhibitor of phosphatidylinositol 3-kinase (PI3K), was purchased from Beyotime. The cells were pretreated with LY294002 (50 μM) for 1 h. The siRNA sequences for target genes are listed in Table 1.

### 2.2. Transwell Assay

The treated-cell suspension (200 μL) in a serum-free medium was added to a Transwell chamber (8 μm; BD Biosciences, San Jose, CA, USA), with or without Matrigel coating. Then, 500 μL of medium containing 10% FBS was added to the lower chamber. After 24 or 48 h, cotton swabs were used to remove cells from the upper chamber. Cells were fixed with 4% paraformaldehyde and incubated with crystal violet. Stained cells were counted at 40× magnification in five randomly selected files.

### 2.3. Western Blotting

Total cell extracts were harvested and solubilized in the radioimmunoprecipitation assay (RIPA) lysis buffer (Solarbio, Beijing, China). Nuclear proteins were collected using a Nucleoprotein Extraction Kit (Keygen Biotech, Nanjing, China). The concentration of the lysate was detected using a bicinchoninic acid (BCA) kit (Thermo Scientific, Rockford, IL, USA). Then, proteins (30 μg) were separated by 10% sodium dodecyl sulfate–polyacrylamide gel electrophoresis (SDS–PAGE) and transferred onto a 0.2 μm polyvinylidene fluoride (PVDF)membrane (Millipore). After blocking with 5% nonfat milk, the membranes were incubated with the corresponding antibodies at 4 °C overnight. The antibodies used are listed in Table 2. The next day, the membrane was incubated with a secondary antibody (1:10,000; Solarbio, Beijing, China) for 1 h. Western blot bands were tested using the Chemidoc™ Touch imaging system.

### 2.4. Chromatin Immunoprecipitation (ChIP)

ChIP assay was performed according to a previous report [32]. Briefly, the treated cells were cross-linked with 1% formaldehyde, and the cross-linking reaction was quenched with10x glycine. The cells were lysed using the SDS lysis buffer. Lysates were immunoprecipitated with the anti-Snail antibody. After precipitation with protein A/G agarose beads, the DNA cross-linked with Snail was obtained and subjected to a polymerase chain reaction (PCR) with the following primers: antisense: 5′-ACTCCAGGCTAGAGGGTCACC-3′; sense:5′-CCGCAAGCTCACAGGTGCTTTGCAGTTCC-3′.

### 2.5. Immunofluorescence

PANC-1 cells were seeded on coverslips in a 24-well plate. Aftertreatment, the coverslips were fixed, permeabilized, blocked for 1 h with 10% goat serum, and incubated with anti-E-cad, anti-VIM, anti-N-cad, and anti-Snail (1:200) antibodies overnight at 4 °C. Next, the coverslips were stained with fluorescein isothiocyanate (FITC)-labeled secondary antibodies at room temperature for 1 h. Nuclei were marked with 4′,6-diamidino-2-phenylindole dihydrochloride (DAPI; 1:5000). The coverslips were washed three times with phosphate-buffered saline containing 0.05% Tween 20 and photographed using a laser scanning microscope.

### 2.6. Wound-Healing Assays

Treated cells were seeded in a 6-well plate. When the cell confluence was close to 100%, two straight lines were drawn in the 6-well plate with a 10 μL pipette tip and washed with PBS to remove floating cells. They were then incubated with a serum-free medium with 5% CO_2_ at 37 °C. Images were captured under a 40x inverted microscope at 0, 24, and 48 h to calculate the scratch-healing rate.

### 2.7. Real-Time PCR

Total RNA was extracted using the Total RNA Extraction Kit (Promega, Madison, WI, USA) according to the manufacturer’s instructions. Then, it was reverse-transcribed into cDNA using a HiFiScript first-strand cDNA synthesis kit (CWBIO, Beijing, China).Real-time PCR was performed using ChamQTM Universal SYBR qPCR Master Mix (CWBIO, Beijing, China) and detected using an Applied Biosystems7500 Fast Dx Real-time PCR Instrument (Thermo Fisher Scientific, Rockford, IL, USA).The relative mRNA expression was calculated using the 2^−ΔΔCt^ method. The primer sequences used in this study are listed in Table 3.

### 2.8. Flow Cytometry Analysis and Sorting

Treated cells were harvested in centrifuge tubes, washed with PBS, and blocked for 1 h with 5% FBS. Then, the cells were culturedwithanti-CD24-PE (0.2 μg/10^6^ cells; Abcam, Cambridge, UK), and anti-CD44-FITC (2 μg/10^6^ cells; Abcam, Cambridge, UK) at 37 °C for 30 min in the dark. Analysis was performed using a FACS Calibur cytometer (BD, Biosciences, San Jose, CA, USA). The CD24^+^/CD44^+^-PANC-1 cells were sorted using a BD FACSAria II cell sorter (Biosciences, San Jose, CA, USA) and lysed with the RIPA lysis buffer for Western blotting analysis.

### 2.9. Adherent Assay

PANC-1 cells were seeded in a 24-well plate. After 3 h of incubation, the number of adherent cells was counted and the percentage of adherent cells was calculated as follows:(adherent cells/total seeded cells) × 100%.

### 2.10. Colony Formation Assay

PANC-1 cells were inoculated into a 6-well plate at 10^3^ cells/well and cultured in an incubator with 5% CO_2_ at 37 °C for 7–14 d. PANC-1 cells were fixed in anhydrous methanol for 15 min and stained with Giemsa solution (Solarbio, Beijing, China) at room temperature for 20 min. Then, the Giemsa solution was discarded, and the cells were washed with PBS. The colony formation rate was calculated as follows: (number of cells with clone spheres/number of inoculated cells) × 100% [33].

### 2.11. Statistical Analysis

Statistical analyses were conducted with software SPSS version 16.0. Data obtained from three independent experiments are expressed as the mean ± standard deviation (SD). Experimental and control groups were compared using a Student’s *t*-test and analysis of variance. Statistical significance was set at * p < 0.05.

## 3. Results

### 3.1. Enhanced Migration and Invasion Abilities Induced by COL11A1 in Pancreatic Cancer Cells

**Figure 1:**
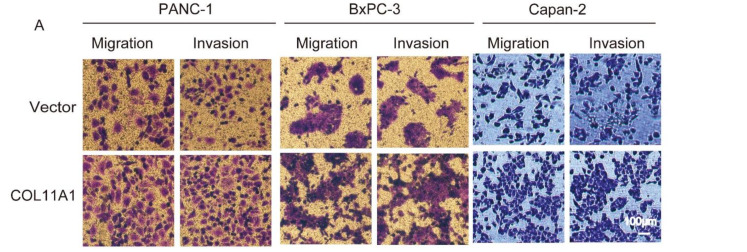

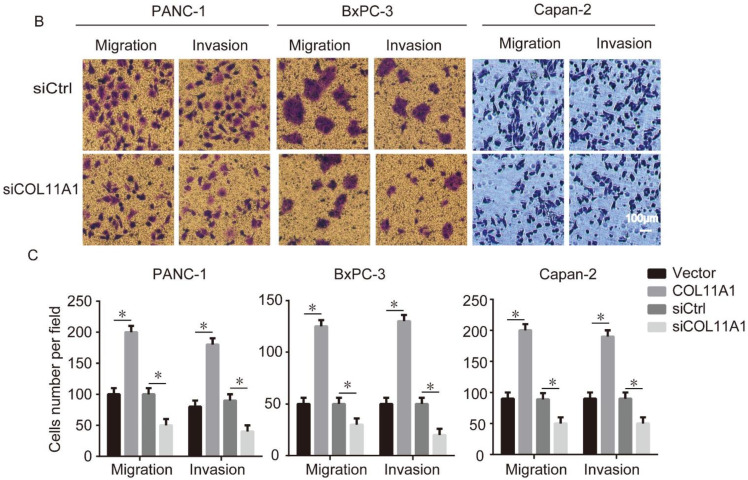
Collagen type XI α1 (COL11A1) promoted the migration and invasion abilities of pancreatic cancer cells: (**A**) overexpression of COL11A1 induced by pCMV3-COL11A1 promoted the migration and invasion of Capan-2, BxPC-3, and PANC-1 cells; (**B**) small interfering (si)-COL11A1 inhibited the migration and invasion of PANC-1, BxPC-3, and Capan-2 cells; (**C**) the number of invasive and migrated pancreatic cancer cells treated with pCMV3-COL11A1 or siCOL11A1 (*n* = 3, * *p* < 0.05).

Several reports have indicated thatCOL11A1 is overexpressed in pancreatic cancer tissues [34,35]. Our previous studies also confirmed that COL11A1 is highly expressed in pancreatic cancer cells and associated with apoptosis and gemcitabine resistance [6]. To further explore whether COL11A1 regulates the invasion and migration of pancreatic cancer cells, COL11A1 was highly expressed in BxPC-3, PANC-1, and Capan-2 cells transfected with pCMV3-COL11A1. Transwell assay results illustrated that high COL11A1 expression markedly enhanced the invasion and migration of pancreatic cancer cells (Figure 1A). Conversely, after COL11A1 was silenced in BxPC-3, Capan-2, and PANC-1 cells using siCOL11A1, the invasion and migration abilities of the pancreatic cancer cells were significantly suppressed (Figure 1B). Additionally, we examined the transfection efficiency of pCMV3-COL11A1 and siCOL11A1 in PANC-1 cells by Western blotting, the results are shown in Supplementary Figure S1A. All data indicate that COL11A1 contributes to the invasion and migration abilities of pancreatic cancer cells.

### 3.2. COL11A1 Modulated the EMT-likePhenotypic Changes and Matrix Metalloproteinase (MMP)-2/9Expression Levels

**Figure 2:**
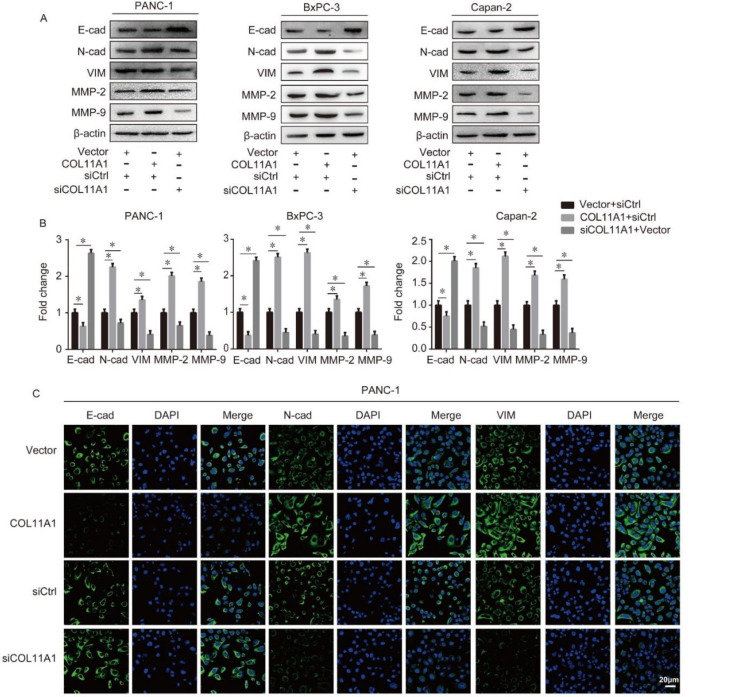

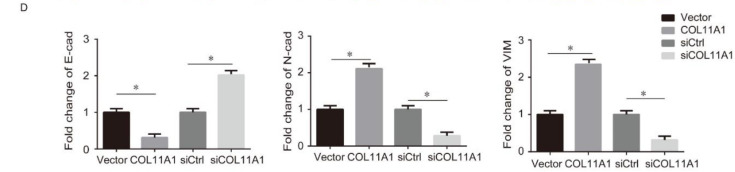
COL11A1 modulated the expression levels of epithelial-mesenchymal transition (EMT) markers and matrix metalloproteinase (MMP)-2/9: (**A**) expression levels of EMT markers and MMP-2/9 were detected by Western blotting analysis in BxPC-3, Capan-2, and PANC-1 cells transfected with siCOL11A1 or pCMV3-COL11A1; (**B**) protein levels of EMT markers and MMP-2/9 were normalized to those of β-actin in PANC-1, BxPC-3, and Capan-2 cells after the indicated treatment; (**C**) expression levels of EMT markers were examined by confocal microscopy in PANC-1 cells transfected with pCMV3-COL11A1 or siCOL11A1 (×100); (**D**) expression levels of N-cadherin (N-cad), E-cadherin (E-cad), and vimentin (VIM) were calculated as a ratio compared with the controls (*n* = 3, * *p* < 0.05).

To examine the underlying mechanism by which COL11A1 promotes the invasion and migration of pancreatic cancer cells, we measured the expression levels of EMT markers and MMP-2/9 using Western blotting and immunofluorescence. Western blotting results revealed that pCMV3-COL11A1-transfection upregulated the expression levels of N-cad, VIM, and MMP-2/9 but reduced the expression of E-cad, in BxPC-3, Capan-2, andPANC-1 cells. In addition, the knockdown of COL11A1 caused the opposite result (Figure 2A,B). To further confirm the above results, we measured the expression levels of EMT markers (N-cad, E-cad, and VIM) using an immunofluorescence assay. The results were consistent with the findings of Western blotting; COL11A1 overexpression increased the expression levels of VIM and N-cad and reduced the expression of E-cad in PANC-1 cells (Figure 2C,D). According to the above experimental results, we speculated that COL 11A1 promoted migration and invasion by modulating the EMT and MMP-2/9, especially in PANC-1 cells, so we selected PANC-1 cells to perform the following experiments.

### 3.3. COL11A1 Induced the Migration and Invasion of Pancreatic Cancer Cells via the Activation of theAKT/GSK-3β/Snail Pathway

**Figure 3:**
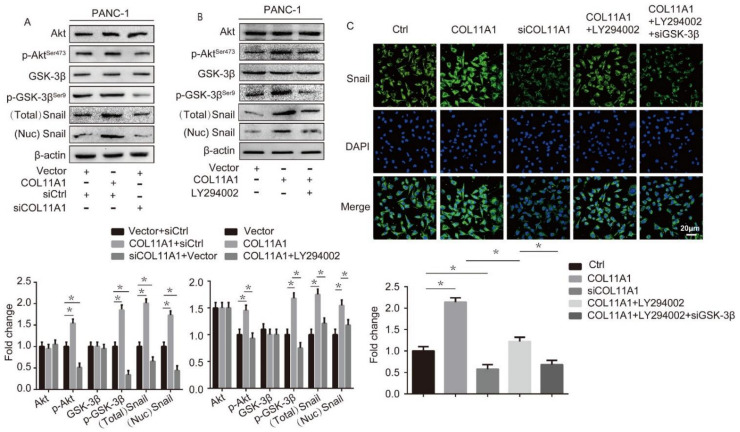
COL11A1 activated the serine-threonine kinase (AKT)/glycogen synthase kinase (GSK)-3β/Snail signaling pathway: (**A**) expression levels of AKT, GSK-3β, phosphor (p)-AKT^Ser473^, p-GSK-3β^Ser9^, and Snail in PANC-1 cells transfected with pCMV3-COL11A1 or siCOL11A1 were measured by Western blotting; (**B**) Western blotting results showing the expression levels of AKT, GSK-3β, p-AKT^Ser473^, p-GSK-3β^Ser9^, and Snail in PANC-1 cells treated with pCMV3-COL11A1 and LY294002; (**C**) immunofluorescence analysis of Snail expression by confocal microscopy in PANC-1 cells transfected with pCMV3-COL11A1, siCOL11A1, and/or LY294002, siGSK-3β(×100) (*n* = 3, * *p* < 0.05).

**Figure 4:**
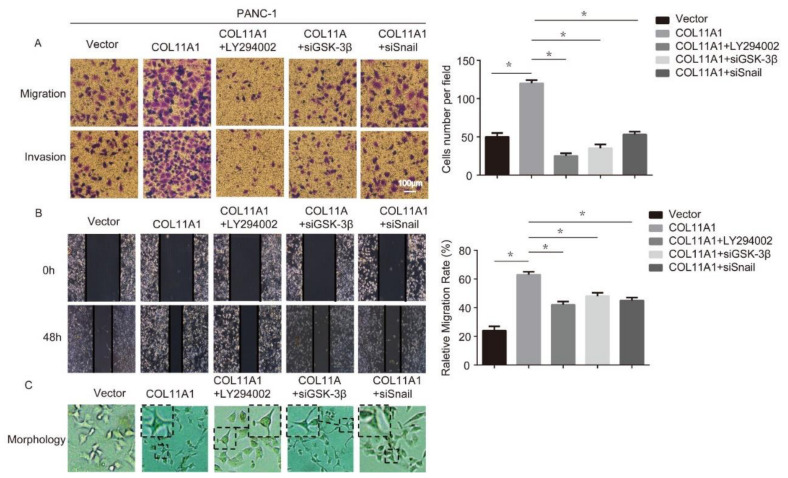
COL11A1 promoted the migration and invasion of pancreatic cancer cells via the Akt/GSK-3β/Snail signaling pathway: (**A**) transwell assay was performed to detect the migration and invasion abilities of PANC-1 cells after treatment; (**B**)the migration ability of treated PANC-1 cells was tested using a wound-healing assay; (**C**) morphology of PANC-1 cells after various treatments. (*n* = 3, * *p* < 0.05).

Several studies have suggested that COL11A1 leads to the activation of the AKT signaling pathway in various cellular events. Our previous study demonstrated that COL11A1 can phosphorylate AKT in pancreatic cancer cells [6]. AKT is an essential pathway associated with EMT and plays a key role in cell proliferation and migration [36]. Furthermore, AKT/GSK-3β/Snail signaling is involved in the migration and invasion of hepatocellular carcinoma cells [27]. Therefore, we tested the modulation effect of COL11A1 on AKT/GSK-3β/Snail signaling using Western blotting in PANC-1 cells. The results demonstrated that COL11A1 promoted the phosphorylation of AKT^Ser473^ and GSK-3β^Ser9^ and simultaneously enhanced the expression and nuclear localization of Snail (Figure 3A). Furthermore, Western blotting results also illustrated that LY294002 weakened the function of COL11A1 in AKT/GSK-3β/Snail signaling (Figure 3B). To further confirm that COL11A1 regulates the nuclear localization of Snail by activating the AKT/GSK-3β pathway in PANC-1 cells, we examined the expression levels of Snail using immunofluorescence. The results demonstrated that COL11A1 increased the nuclear expression levels of Snail in PANC-1 cells, whereas LY294002 and siGSK-3β inhibited the effect of COL11A1 on the nuclear localization of Snail (Figure 3C). To prove that COL11A1 induced cell invasion and migration via the AKT/GSK/Snail signaling pathway, we performed wound healing and transwell assays. The results showed that LY294002, siGSK-3β, and siSnail inhibited the role of COL11A1 in the invasion and migration of PANC-1 cells (Figure 4A,B). During the cultivation of PANC-1 cells transfected with pCMV3-COL11A1, we found that these cells exhibited a more spindle-like cell shape with more pseudopods (Figure 4C), indicating that COL11A1 might mediate morphological changes with EMT features in tumor cells. Therefore, COL11A1 promotes migration and invasion by activating the AKT/GSK-3β/Snail pathway.

### 3.4. AKT/GSK-3β/Snail Axis Was Pivotal for COL11A1-Induced EMT

**Figure 5:**
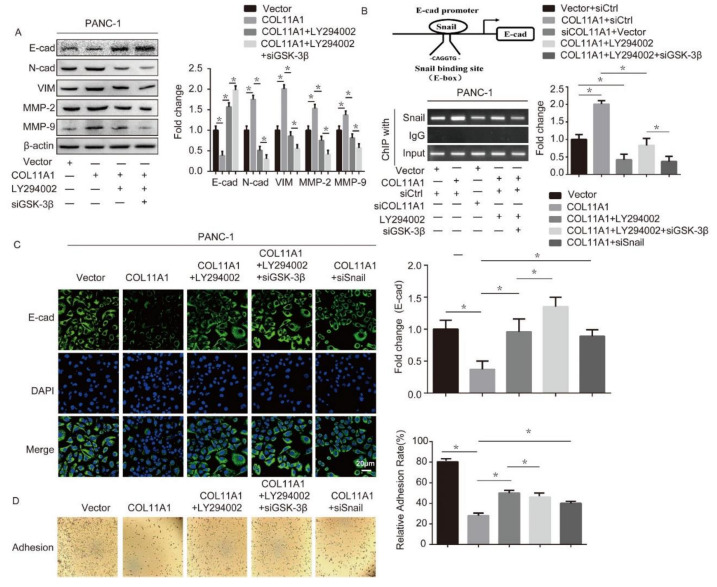
COL11A1 promoted EMT via the AKT/GSK-3β/Snail signaling pathway: (**A**) Western blotting results showing the expression levels of EMT markers and MMP-2/9 in PANC-1 cells transfected with pCMV3-COL11A1, LY294002, and siGSK-3β; (**B**) the sequence and position of the Snail binding site in the E-cad promoter are shown. Chromatin immunoprecipitation (ChIP) of Snail in the E-cad promoter. PANC-1 cells were transfected withpCMV3-COL11A1, siCOL11A1, siGSK-3β, and LY294002; (**C**) immunofluorescence analysis of E-cad expression levels in PANC-1 cells transfected with pCMV3-COL11A1, LY294002, siGSK-3β, and siSnail (×100); (**D**) the images show the adhesion ability of the indicated cells after different treatments. (*n* = 3, * *p* < 0.05).

To further verify that COL11A1 promoted the EMT process in pancreatic cancer cells by activating the AKT/GSK-3β/Snail signaling pathway, we first detected the expression levels of E-cad, N-cad, VIM, and MMP-2/9 in PANC-1 cells with different treatments by Western blotting. We found that compared with the COL11A1 alone treatment group, the expression of E-cad was significantly upregulated, while the expression levels of N-cad, VIM, and MMP-2/9 were downregulated in the LY294002, siGSK-3β, and COL11A1 co-treatment group (Figure 5A). Studies have shown that Snail binds to the E-cad promoter, inhibiting the transcription of E-cad and contributing to the occurrence of EMT [37]. Therefore, we investigated the effect of COL11A1 on the binding efficacy of Snail and E-cad using a ChIP assay. The results showed that COL11A1 enhanced Snail/E-cad binding, while siCOL11A1 inhibited it. Furthermore, after the blockage of two downstream effectors, AKT and GSK-3β, the function of COL11A1 in promoting Snail/E-cad binding weakened (Figure 5B). We further confirmed that COL11A1 affected the expression of E-cad via the AKT/GSK-3/Snail signaling pathway by an immunofluorescence assay (Figure 5C). Subsequently, the adhesion abilities of PANC-1 cells treated with different treatments were evaluated. The results suggested that AKT, GSK-3β, and Snail are necessary for COL11A1 to promote the motility and adhesion of PDAC cells (Figure 5D). Therefore, the above results indicate that COL11A1 promotes EMT via AKT/GSK-3β/Snail signaling.

### 3.5. COL11A1 Modulated the Cell Stemness Efficiency by Regulating the AKT/GSK-3β/Snail Signaling Pathway

**Figure 6:**
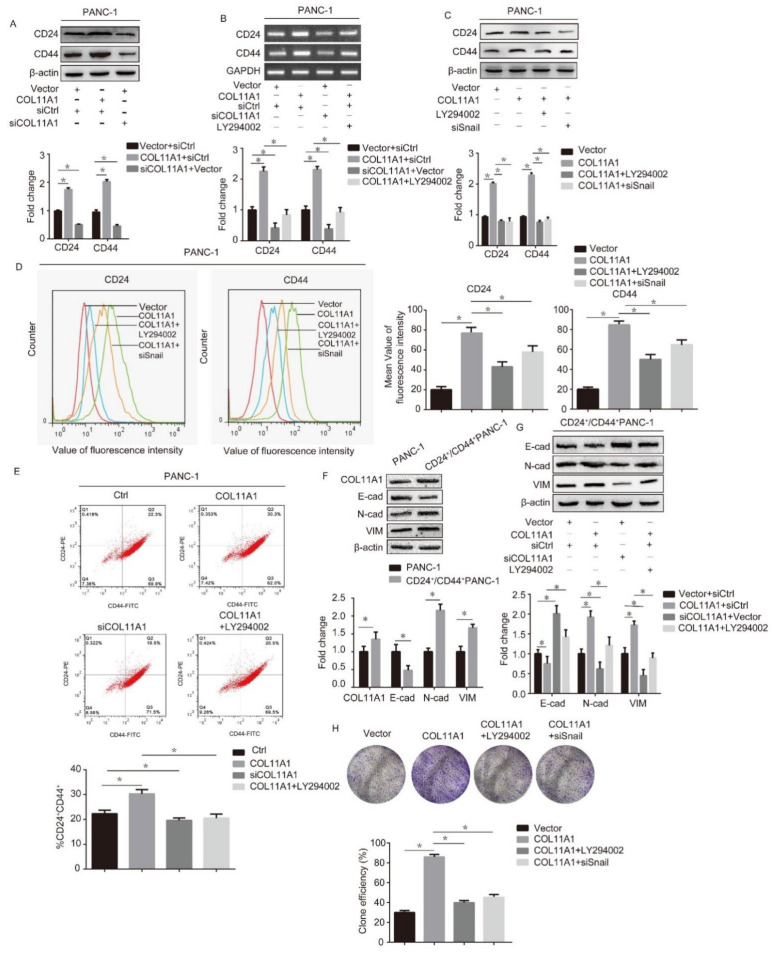
COL11A1 enhanced the cell stemness via the AKT/GSK-3β/Snail signaling pathway: (**A**) Western blotting analysis of cluster of differentiation (CD)-24 and CD44expression levels in PANC-1 cells treated with pCMV3-COL11A1 or siCOL11A1; (**B**) mRNA expression levels of CD24 and CD44 were detected in PANC-1 cells treated with different treatments using real-time polymerase chain reaction (PCR); (**C**) protein expression levels of CD24 and CD44 in PANC-1 cells transfectedwithpCMV3-COL11A1, LY294002, and siSnail; (**D**) PANC-1 cells were treated under different conditions and the expression levels of CD24and CD44 on the cell membranes were detected using flow cytometry; (**E**) flow cytometry was used to detect the percentage ofCD24^+^/CD44^+^ cells among PANC-1 cells treated with different treatments; (**F**) Western blotting was used to detect the expression levels of COL11A1 and EMT markers in PANC-1 and CD24^+^/CD44^+^-PANC-1 cells; (**G**) expression levels of EMT markers in CD24^+^/CD44^+^-PANC-1 cells treated with different treatments; (**H**) colony formation assay was performed in PANC-1 cells with different treatments. (*n* = 3, * *p* < 0.05).

Previous studies have shown that both Snail and E-cad are related to cell stemness [38,39], and our research further confirmed that COL11A1 affects the expression levels of Snail and E-cad in pancreatic cancer cells. Therefore, we suspected that COL11A1 might be associated with cell stemness. To address this possibility, we first detected the function of COL11A1 in the expression of cancer stem cell (CSC)-associated markers (cluster of differentiation (CD)-24 and CD44) in PDAC using Western blotting. The results demonstrated that COL11A1 significantly promoted, while siCOL11A1 inhibited, the expression levels of CD24 and CD44 proteins (Figure 6A). In addition, LY294002 and siSnail weakened the effect of COL11A1 on the mRNA and protein expression levels of CSC-associated markers (Figure 6B,C). Flow cytometric analysis confirmed that COL11A1 promoted the expression levels of CD24 and CD44 on the cell surface, while LY294002 and siSnail blocked these functions (Figure 6D). In addition, flow cytometric analysis showed a significantly higher CD24^+^/CD44^+^ ratio in pCMV3-COL11A1-transfected PANC-1 cells than the untreated cells, and siCOL11A1 and LY294002 weakened COL11A1 function (Figure 6E). To further verify the link between EMT and cell stemness, we detected COL11A1 and EMT marker protein levels in PANC-1 and CD24^+^/CD44^+^-PANC-1 cells by Western blotting. The results illustrated that COL11A1, N-cad, and VIM were significantly expressed in CD24^+^/CD44^+^-PANC-1 cells, while E-cad expression was reduced (Figure 6F). Moreover, in CD24^+^/CD44^+^-PANC-1cells, COL11A1 enhanced the expression levels of N-cad and VIM and simultaneously reduced E-cad expression, while LY294002 inhibited the effect of COL11A1 on CD24^+^/CD44^+^-PANC-1 cells (Figure 6G). These results indicate that the EMT process is more prominent in CD24^+^/CD44^+^-PANC-1 cells and that the COL11A1/AKT/GSK-3β/Snail pathway might effectively manage this process in these cells. In addition, we performed a colony formation assay to evaluate the effect of COL11A1 on the stemness of pancreatic cancer cells. The results showed that more cell spheres, larger microsphere diameters, and higher monoclonal rates were present in the COL11A1 group than the vector group, and the LY294002 and siSnail groups reversed the effect induced by COL11A1 (Figure 6H). Therefore, COL11A1 enhances the stemness of pancreatic cancer cells by activating the AKT/GSK-3β/Snail signaling pathway.

## 4. Discussion

PDAC is characterized by the considerable accumulation of extracellular matrix (ECM) components. Studies have shown that collagen (COLI, COLIII, and COLIV) in the ECM component is closely associated with the metastatic process [40]. Many elaborate mechanistic studies have demonstrated that collagen drives tumorigenesis [41,42]. However, the specific collagen factor involved in the pathogenesis of ECM-mediated metastasis in PDAC remains unknown. Compared with other investigated collagen factors, collagen XI/COL11A1 is scarce in normal pancreatic tissues. Therefore, abnormal expression of COL11A1 during cancer development might constitute a specific indicator of neoplastic transformation [43]. In the current study, we showed that COL11A1 enhanced the invasion and migration of pancreatic cancer cells and promoted the EMT process and cell stemness. Our results demonstrate that COL11A1 might be a potential indicator for diagnosing PDAC metastasis at an early stage.

Previous studies have shown that COL11A1 mediates Akt^Ser473^ activation via integrin α1β1 and discoid in domain receptor 2 to implement signal transduction in a variety of biological events, including migration and invasion [6,16,19]. p-Akt^Ser473^ inactivates GSK-3βby phosphorylating the ser-9 site, while p-GSK-3β^Ser9^ increases the nucleation of Snail, an inhibitor of E-cad. In sequence, p-GSK-3β^Ser9^/Snail manages the EMT process by regulating the epithelial and mesenchymal genes in epithelial tumors [44,45]. In this study, we found that COL11A1 promoted the phosphorylation of AKT^Ser473^ and GSK-3β^Ser9^ and then enhanced both the expression and nuclear localization of Snail. GSK-3β acts as the main kinase that phosphorylates Snail, thereby facilitating its degradation via the ubiquitin–proteasome pathway [46]. We proposed that the COL11A1/AKT-mediated inactivation of GSK-3β was responsible for the cellular accumulation and elevated nuclear localization of Snail. In turn, Snail transcriptionally suppressed the expression of E-cad, which is a master modulator of the epithelial phenotype. Moreover, the abnormal regulation of E-cad leads to a mesenchymal-like phenotype in infiltrated carcinoma cells. Therefore, by modulating the alteration of associated markers, COL11A1 generates the AKT/GSK-3β/Snail-dependent EMT program and promotes cell migration/invasion potency in PDAC. Our study identified the elaborate mechanism by which the COL11A1/AKT/GSK-3β/Snail cascade is potentially involved in PDAC metastasis. We found that Snail acts as a fundamental factor in molecular transduction, by which COL11A1 manipulates tumor cell infiltration. Consequently, we indicated that a therapeutic measure capable of blocking Snail should efficiently inhibit EMT and limit the invasive process in PDAC with high COL11A1 expression. In future studies, it would be interesting to investigate the functions of contextual signals in implementing EMT programs in the PDAC TME.

The association between cell stemness and EMT has attracted the attention of many researchers due to the similar roles that these two processes play in cancer cell metastasis. Evidence suggests that epithelial CSCs express many mesenchymal markers that are related to advanced malignant features, including relapse, invasiveness, and metastatic dissemination [47]. Recent evidence indicates that cells undergoing EMT acquire cancer stem-like properties, resulting in the development of many cancers [48]. E-cad knockdown induces a CSC-like phenotype and drug resistance in cancer cells [39]. Snail ablation attenuates colony formation and weakens the expression of CSCs markers [49]. CD24^+^/CD44^+^ and CD133^+^CXCR4^+^ cells have been proposed to represent CSCs in PDAC, based on their self-renewal ability [50]. PANC-1 cells positively expressed the CD24^+^/CD44^+^ cell surface phenotype [51], so we chose them to observe the effects of COL11A1/AKT/GSK-3β/Snail signaling on both EMT and cell stemness processes. Our study provides further evidence that both high expressed-COL11A1 and abnormal EMT markers are observed in CSCs of PDAC.

We elucidated the mechanism by whichCOL11A1regulated the expression levels of both mRNAs and proteins involved in EMT and stem cell-like properties, such as enhanced colony formation. The CD24^+^/CD44^+^ cells with high COL11A1 expression may undergo EMT.COL11A1 is absent in normal pancreatic tissues, which may be a crucial factor for distinguishing between cancer initiation and development. Therefore, we propose that COL11A1 inhibitors have high therapeutic potential in simultaneously affecting EMT/CSC properties in pancreatic cancer, thereby suppressing the migration and invasion of cells, which provides an additional means for implementing conventional therapy. Additionally, COL11A1 plays a pivotal role in manipulating the cell stemness and EMT, essentially serving as a link between the two processes. Therefore, the molecular mechanisms underlying the effect of COL11A1 on EMT require further elucidation.

## 5. Conclusions

In summary, our study demonstrated that COL11A1 effectively induced EMT progression and cell stemness by activating the AKT/GSK-3β/Snail signaling pathway to enhance the migration and invasion abilities of pancreatic cancer cells. The present study revealed a novel signal transduction cascade, in which COL11A induced EMT and cell stemness, and highlighted a potential therapeutic target for the metastasis of PDAC.

## Figures and Tables

**Table 1:** Sequences of siRNAs.

Gene	siRNA Sequences (5′ to 3′)
siCOL11A1-1	5′-CUCCAGUUGAUGUACUAAATT-3′
siCOL11A1-1	5′-CCAGAGGAUAUAAUCGAAUTT-3′
siSnail-1	5′-GCGAGCUGCAGGACUCUAA-3′
siSnail-2	5′-GUGACUAACUACUGCAAUAA-3′
siGSK-3β-1	5′-GGGCCUUAUAUACUCUAAA-3′
siGSK-3β-2	5′-GCCUCAAAGUAGUCCAUAU-3′

**Table 2:** Antibodies used for western blotting analysis.

Name	Company	Catalog Number	Antibody Concentration
E-cad	Cell Signaling Technology, Massachusetts USA	14472S	1:1000
N-cad	Cell Signaling Technology, USA	13116S	1:1000
VIM	Cell Signaling Technology, USA	5741S	1:1000
Snail	Cell Signaling Technology, USA	3879S	1:1000
AKT	Proteintech, Chicago, USA	60203-2-Ig	1:2000
p-AKT^Ser473^	Cell Signaling Technology, USA	4060S	1:2000
MMP-2	Cell Signaling Technology, USA	40994S	1:1000
MMP-9	Cell Signaling Technology, USA	13667S	1:1000
GSK-3β	Cell Signaling Technology, USA	12456S	1:1000
p-GSK-3β^Ser9^	Cell Signaling Technology, USA	5558S	1:1000
CD24	Proteintech, USA	18330-1-AP	1:500
CD44	Proteintech, USA	60224-1-Ig	1:1000
β-actin	Sungene Biotech, Tianjin, China	KM9001	1:5000

**Table 3:** Primer sequences used for real-timepolymerase chain reaction (PCR) analysis.

Genes	Primer Sequences
*CD24*	F: 5′-TGCTCCTACCCACGCAGATT-3′R: 5′-GGCCAACCCAGAGTTGGAA-3′
*CD44*	F: 5′-CACAATCCAGGCAACTCCTA-3′R: 5′-TACTCTGCTGCGTTGTCATT-3′
*GAPDH*	F: 5′-TGCACCACCAACTGCTTAGC-3′R: 5′-GGCATGGACTGTGGTCATGAG-3′

## Data Availability

Not applicable.

## Supplementary Materials

The following supporting information can be downloaded at: https://www.mdpi.com/article/10.3390/biom12030391/s1. Figure S1: Transfection efficiency of COL11A1 plasmid and siRNA, and the graphical abstract of this paper.
