# Supplementary figures and images for: COL11A1-Driven Epithelial–Mesenchymal Transition and Stemness of Pancreatic Cancer Cells Induce Cell Migration and Invasion by Modulating the AKT/GSK-3β/Snail Pathway
Source: Biomolecules. 2022 Mar 2;12(3):391. doi: 10.3390/biom12030391 (PMC8945532; doi:10.3390/biom12030391)

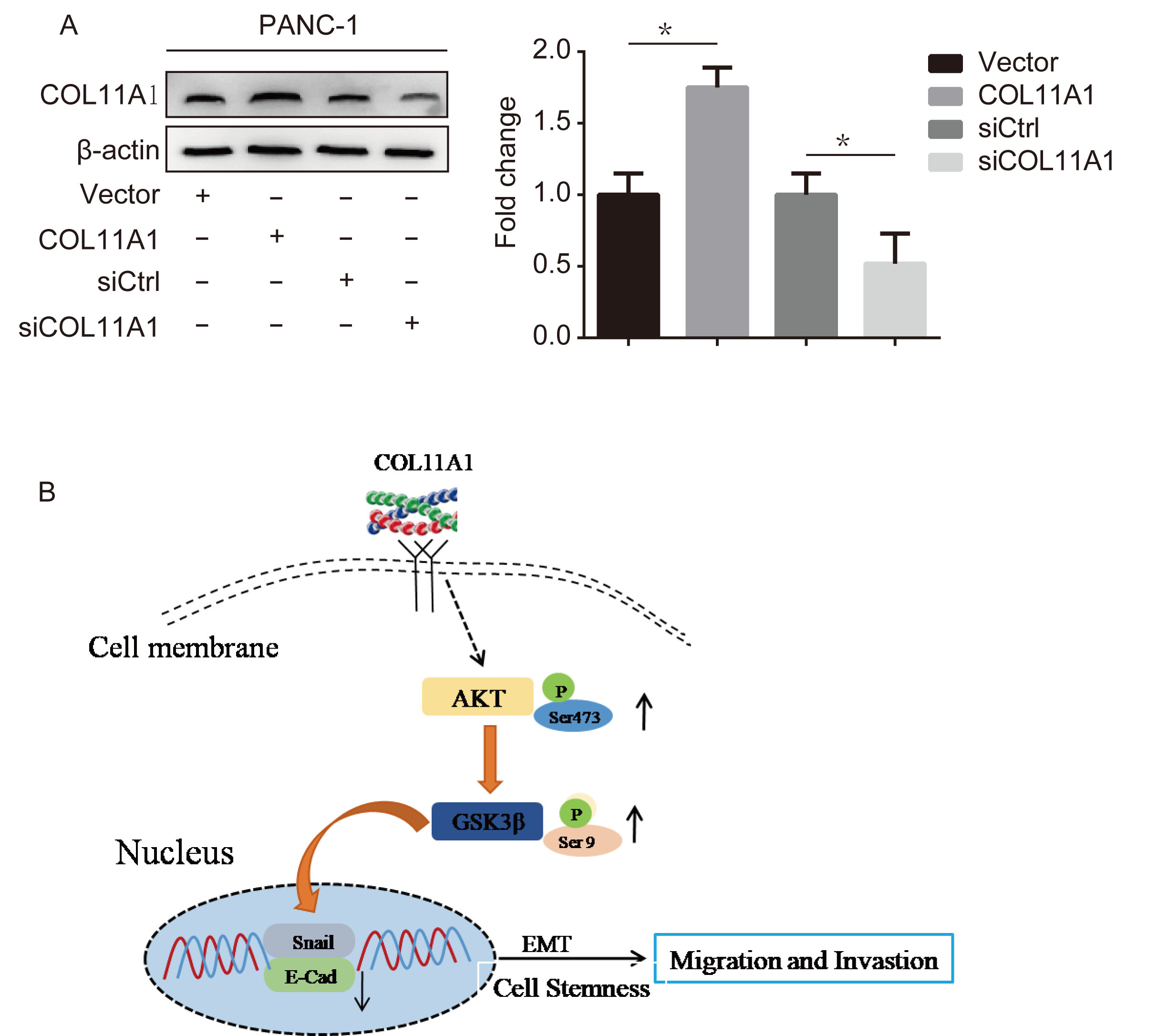

Supplement: Supplementary file 1 [file biomolecules-12-00391-s001.zip › biomolecules-1572578-supplementary.jpg]
